# *Journal of Hematology & Oncology* reviewer acknowledgement 2013

**DOI:** 10.1186/1756-8722-7-12

**Published:** 2014-02-05

**Authors:** Delong Liu

**Affiliations:** 1 New York Medical College, New York, USA

## Abstract

**Contributing reviewers:**

The editors of *Journal of Hematology & Oncology* would like to thank all our reviewers who have contributed to the journal in Volume 6 (2013).

## 

Seema Agarwal

United States of America

Akintunde Akinleye

United States of America

Donatella Aldinucci

Italy

Robert Amato

United States of America

Ningfei An

United States of America

Peter Anderson

United States of America

Rami Aqeilan

Israel

Philippe Arnaud

France

Francis Ayuk

Germany

Jeff Barrett

United States of America

Surinder Batra

United States of America

Paul Beaudry

Canada

Leonidas Benetatos

Greece

Paolo Bernasconi

Kazakhstan

Zwi Berneman

Belgium

Jaap Jan Boelens

Netherlands

Attilio Bondanza

Italy

Elisa Boscolo

United States of America

Joseph Brandwein

Canada

Adam Brufsky

United States of America

Brad Bryan

United States of America

Nicholas Burgis

United States of America

Thomas Burmeister

Germany

Alan Burnett

United Kingdom

Amanda Cashen

United States of America

Richard Cathomas

Switzerland

Ling Cen

United States of America

Hong Chang

Canada

Nan-Shan Chang

Taiwan

Changyan Chen

United States of America

Liguang Chen

United States of America

Liang Cheng

United States of America

Alexander Chi

United States of America

Andy Chien

United States of America

Dai Chihara

Japan

William Cho

Hong Kong

Michael Chopp

United States of America

Magdalena Chrzanowska-Wodnicka

United States of America

Shih-Sung Chuang

Taiwan

Fabrice Cognasse

France

Gaetano Corazzelli

Italy

Mei-Zhen Cui

United States of America

Nico Dantuma

Sweden

Evandro De Azambuja

Belgium

Christophe De Romeuf

France

Adriana De Siervi

Argentina

Paolo Dion

Italy

Angela Dispenzieri

United States of America

Jixin Dong

United States of America

Caigan Du

Canada

James Eddy

United States of America

Stephan Ensminger

Germany

Mehmet Ali Erkurt

Turkey

Beth Faiman

United States of America

Marco Falasca

United Kingdom

Isabella Faraoni

Italy

Ru Feng

China

Michael Flister

United States of America

Alessandro Fornari

Italy

Nathan Fowler

United States of America

Massimo Franchini

Italy

Arnold Freedman

United States of America

Christopher French

United States of America

Stefan Fröhling

Germany

Teruhiko Fujii

Japan

Yutaka Fujiwara

Japan

Seiji Fukuda

Japan

Shirish Gadgeel

United States of America

Gianluca Gaidano

Italy

Peter Gaines

United States of America

Kristen Ganjoo

United States of America

Christian Geisler

Denmark

Jean-Pierre Gerard

France

Laurence Gerard

France

Herve Ghesquieres

France

Stephane Giraudier

France

Stefania Gobessi

Italy

Abhijit Godbole

United States of America

Josee Golay

Italy

Steven Gore

United States of America

Andre Goy

United States of America

Richard Greil

Austria

Baiwei Gu

United States of America

Göhring Gudrun

Germany

Angelo Guerrasio

Italy

Thomas Habermann

United States of America

Michiyuki Hakozaki

Japan

Maria Francisca Ham

Indonesia

Tanja Hartmann

Austria

Robert Hasserjian

United States of America

Aiwu He

United States of America

Charles Hemenway

United States of America

Vera Hirsh

Canada

Georg Hopfinger

Austria

Chung-Tsen Hsueh

United States of America

Jiong Hu

China

He Huang

China

Liang Huang

United States of America

Yujie Huang

United States of America

Sara Hurvitz

United States of America

Shinsuke Iida

Japan

Martino Introna

Italy

Fumihiro Ishida

Japan

Yasuo Iwadate

Japan

Jun Iwashita

Japan

Elias Jabbour

United States of America

Tang-Her Jaing

Taiwan

Gaetan Jego

France

Yuxia Jia

United States of America

Enzi Jiang

United States of America

Yongping Jiang

China

Jie Jin

China

Liang Jin

Australia

Qihuang Jin

United States of America

Grace Kao

United States of America

Sabine Kasimir-Bauer

Germany

Masaru Katoh

Japan

Chul Geun Kim

Korea, South

Won Seog Kim

Korea, South

Hitoshi Kiyoi

Japan

Wolfram Klapper

Germany

Young Hyeh Ko

Korea, South

Michael Koldehoff

Germany

Hasan Korkaya

United States of America

Andrei Kramerov

United States of America

Eric Kraut

United States of America

Geoffrey Ku

United States of America

Karin Kurnik

Germany

Junya Kuroda

Japan

Mateusz Kurzawski

Poland

Toshihiro Kushibiki

Japan

Intidhar Labidi-Galy

United States of America

Gurpreet Lamba

United States of America

Manuel Lemos

Portugal

Ji-Liang Li

United Kingdom

Tianhong Li

China

Yangqiu Li

China

Zhong Li

China

Sharon Liang

United States of America

Evi Lianidou

Greece

Wolfgang Link

Portugal

Eva Lion

Belgium

Delong Liu

United States of America

Hong Liu

United States of America

Jianguo Liu

United States of America

Cristiana Lo Nigro

Italy

Irene Lorand-Metze

Brazil

Nancy Louis

United States of America

Cynthia Ma

United States of America

Daoxin Ma

China

Xiaotu Ma

United States of America

Anne-Marie Madec

France

Michael Makris

United Kingdom

Elisabet Esteve Manasanch

United States of America

Ferdinando Mannello

Italy

Pier Mannuccio Mannucci

Italy

Tibiletti Maria Grazia

Italy

Ana Marques

United Kingdom

William Matsui

United States of America

Fabrizio Mattei

Italy

Sandra Mcallister

United States of America

Michal Mego

United States of America

Shu Meng

United States of America

Adam Metwalli

United States of America

Stefan Meyer

United Kingdom

Joseph Mikhael

United States of America

Michele Milella

Italy

Ken Mills

United Kingdom

Mark Minden

Canada

Hans Minderman

United States of America

Tony Mok

Hong Kong

David Mole

United Kingdom

Hiroyuki Momota

Japan

Tammy Morrish

United States of America

Hasan Mukhtar

United States of America

Steffan Nawrocki

United States of America

John Nelson

United States of America

Seiji Niho

Japan

Shozo Nishida

Japan

Bodil Norrild

Denmark

Rajesh Kumar NV

United States of America

Massimo Offidani

Italy

Seiichi Okabe

Japan

Yasuhiro Oki

United States of America

Horatiu Olteanu

United States of America

Toru Ouchi

United States of America

Thomas Pabst

Switzerland

Chin-Chen Pan

Taiwan

Helen Papadaki

Greece

Tomas Papajik

Czech Republic

Samir Parekh

United States of America

Upendra Parvathaneni

United States of America

Tejal Patel

United States of America

Swati Pathak

United States of America

Jun Peng

China

Christian Peschel

Germany

Katharina Pfistershammer

Austria

Rocco Piazza

Italy

Hélène A Poirel

Belgium

Guy Pratt

United Kingdom

Vít Procházka

Czech Republic

Soham Puvvada

United States of America

Anne Quillet-Mary

France

Farhang Rabbani

United States of America

Shamudheen Rafiyath

United States of America

Pablo Ramirez

Chile

William Redmond

United States of America

Ruibao Ren

United States of America

Adam Resnick

United States of America

Ryan Riddle

United States of America

Tadeusz Robak

Poland

Jesse Roman

United States of America

Stefano Rosso

Italy

John Ryan

United States of America

Nabil Saba

United States of America

Lorena Sánchez-Martín

Spain

Valeria Santini

Italy

Yorifumi Satou

United Kingdom

Yogen Saunthararajah

United States of America

Kerry Savage

Canada

Nic Savaskan

Germany

Heiner Schäfer

Germany

Christian Scharenberg

Sweden

Mirta Schattner

Argentina

Gary Schiller

United States of America

Bin Shan

United States of America

Mahesh Sharma

United States of America

Anieta Sieuwerts

Netherlands

David Snyder

United States of America

Brigitte Sola

France

Tobias Sperka

Germany

James Spicer

United Kingdom

Daniel Starczynowski

United States of America

Peter Steinberger

Austria

Cordula Stover

United Kingdom

K. Stephen Suh

United States of America

Anders Sundan

Norway

Ritsuro Suzuki

Japan

Arthur Sytkowski

United States of America

Philippe Szankasi

United States of America

Shinichiro Takahashi

Japan

Jin Takeuchi

Japan

Dipti Talaulikar

Australia

Keiji Tanese

Japan

Chih-Hsin Tang

Taiwan

Matthias Tenbusch

Germany

John Thompson

United States of America

Kirsty Thomson

United Kingdom

Kenneth To

Hong Kong

Mariko Tomita

Japan

Naoto Tomita

Japan

Hai Tran

United States of America

Martin Trepel

Germany

Yang Tsai Sheng

Taiwan

William Tse

United States of America

Alessandra Tucci

Italy

Togas Tulandi

Canada

Shinya Uchino

Japan

Henning Ulrich

Brazil

Antonis Valachis

Greece

Ben Valdez

United States of America

Eric Van Den Neste

Belgium

Johan Van Krieken

Netherlands

K. David Voduc

Canada

Maria Teresa Voso

Italy

Domagoj Vucic

United States of America

Christine Walsh

United States of America

Dazhi Wang

United States of America

Donghai Wang

United States of America

Gang Wang

China

Kankan Wang

China

Xin Wang

China

Ya Wang

United States of America

Zilong Wen

Hong Kong

Meir Wetzler

United States of America

Andre Willasch

Germany

Thian-Sze Wong

Hong Kong

Gang Wu

China

Dazhong Xu

United States of America

Qingwen Xu

United States of America

Teng Xu

United States of America

Motoko Yamaguchi

Japan

Ying Yan

United States of America

David Yang

United States of America

Yibin Yang

United States of America

Jean Yared

United States of America

M. James You

United States of America

Ken Young

United States of America

Herbert Yu

United States of America

Bo Yuan

Japan

Zihua Zeng

United States of America

Fenghuang Zhan

United States of America

Chengcheng Zhang

United States of America

Dongwei Zhang

United States of America

Hua Zhang

China

Jiangwei Zhang

United States of America

Jianke Zhang

United States of America

Xiaolei Zhang

United States of America

Xiaowei Zhang

United States of America

Yi Zhang

United States of America

Yuxia Zhang

United States of America

Qian Zhao

China

Zhizhuang Joe Zhao

United States of America

Xiaoyong Zheng

United States of America

Daohong Zhou

United States of America

Zhengping Zhuang

United States of America

Mario Zoratti

Italy

